# Multiple Myeloma Screening Education: A Community-Based Health Literacy Intervention

**DOI:** 10.1158/2767-9764.CRC-26-0056

**Published:** 2026-06-24

**Authors:** Karie Feldman, Hong Li, Diana Basali, Faiz Anwer, Kimberly Bell, Beth Faiman, Raymond D. Jackson, Sandra Mazzoni, Kayona Moore, Natasha Patel, Shahzad Raza, Jason Valent, Heather McKee Hurwitz

**Affiliations:** Cancer Institute, Cleveland Clinic, Cleveland, Ohio.

## Abstract

**Purpose:**

To address the lack of federal screening guidelines and a limited understanding of multiple myeloma among the public and some primary care providers, we created the first community-based multiple myeloma screening program and educational intervention.

**Patients and Methods:**

The academic cancer center partnered with community organizations to distribute an original brochure about multiple myeloma, draw blood to check for monoclonal protein, and survey screening participants about multiple myeloma knowledge and screening experiences. Any Black individual aged 50 or older was eligible. To examine multiple myeloma knowledge retention, study team members surveyed participants after at least 1 week had passed and screening results had been provided. The educational intervention also included continuing medical education (CME) about the early detection of multiple myeloma for healthcare professionals. Analysis utilized descriptive statistics and the McNemar test to assess differences between the initial and follow-up surveys.

**Results:**

From 2023 to 2024, across 22 screening events, 199 participants completed the screening. Among the 158 individuals who completed both surveys, correct responses increased on all items and increased significantly on items about multiple myeloma being a rare cancer of the blood (19%, *P* < 0.0001) and that individuals with monoclonal gammopathy of undetermined significance (MGUS) have an abnormal protein in the blood (12.1%, *P* = 0.002). Screening results identified 21 participants (11%) with MGUS. Forty-six healthcare providers engaged in three CME seminars.

**Conclusions:**

Participants retained knowledge about multiple myeloma gained during the screening. Improved health literacy about cancer risk for multiple myeloma may improve early detection, communication, and decision-making about cancer care.

**Significance:**

This study reveals that community members are receptive to multiple myeloma screening when healthcare professionals communicate with them about their risks and share knowledge about disease symptoms and incidence. Improved health literacy about cancer risk for multiple myeloma may improve early detection, communication, and decision-making about cancer care.

## Introduction

Despite the diagnosis of multiple myeloma in more than 180,000 people worldwide and 30,000 in the United States (1, 2), the general public and primary care providers (PCP) know little about this “hematologic malignancy of antibody-producing plasma cells” (1, 3, 4). One study noted that only 17% of 894 patients diagnosed with multiple myeloma had heard of the condition prior to their diagnosis (5). Lack of knowledge about multiple myeloma contributes to delayed diagnosis; often, both patients and providers attribute multiple myeloma–related symptoms to normal aging or other diseases rather than recognizing the constellation of symptoms associated with multiple myeloma (4, 6, 7). Improving health literacy about multiple myeloma could be particularly impactful for earlier detection, either by requests for testing from patients or by PCPs expanding their clinical acumen to better test for the disease.

Multiple myeloma is preceded by monoclonal gammopathy of undetermined significance (MGUS) and/or smoldering multiple myeloma (SMM; ref. 8). These precursor conditions can be detected with a simple blood test; however, no screening guidelines currently exist. Screening could occur before any signs of illness, but multiple myeloma is typically found only after a patient has an abnormal laboratory test, such as anemia and elevated total proteins, or experiences symptoms such as fatigue, fluid retention with kidney involvement, bone pain, or an unexplained bone fracture. Primary risks of screening include the psychologic stress of positive results, the cost of follow-up, and challenges associated with bone marrow tests (9). Due to these possible burdens, recent studies have recommended identifying those at highest risk as a screening strategy while waiting for results from larger trials that estimate the benefits of randomized screening (9). Nevertheless, the average rate of MGUS to progression of multiple myeloma is 1% per year (10–12). This risk is continuous, and progression risk is assessed in three categories: low risk, intermediate risk, and high risk. For those patients with high-risk MGUS [monoclonal protein (M-protein) >1.5, non–immunoglobulin G (IgG) subtype, and abnormal light chain ratio], the risk of progression is 20% over 20 years (10–12). Similarly, the risk of progression of SMM to multiple myeloma, on average, is 10% per year for the first 5 years (13). In contrast, 50% of patients with high-risk smoldering myeloma (bone marrow plasma cells ≥20%, M-protein >2 g/dL, and free light chain ratio ≥20) can experience progression in the first 2 years (13). Early detection of MGUS or SMM, such as through screening, could lead to life-saving and life-extending benefits with monitoring and early pharmacologic and/or behavioral interventions that may reduce progression to symptomatic multiple myeloma or reduce disease severity (10–16). Although the majority of patients identified with MGUS will never progress to multiple myeloma, some will. Current medical standards suggest that those with MGUS should be followed in case of progression (9).

The prevalence of MGUS is still unclear (17–19), but Black Americans are at threefold risk for the disease compared with White Americans, with people over 50 years old and those with a family history of the disease at the highest risk for developing the condition; research is beginning to demonstrate that these groups may benefit most from screening (9, 20). Although Black individuals make up 13.6% of the US population, new incidence of multiple myeloma among Black Americans is disproportionately higher at about 20% (4). Research has shown that Black patients with multiple myeloma are empowered by increasing their knowledge of multiple myeloma through independent research and that presenting to treating providers as informed patients with multiple myeloma improves their care (14). Unfortunately, most people do not know their family history (21). Screening Black people above 50 years of age is a strategy to detect multiple myeloma early in the highest-risk population.

Especially due to the need for provider and patient education about the nuances of screening for multiple myeloma, we developed the first community-based multiple myeloma screening program for Black individuals above the age of 50. The project sought to improve outcomes for Black individuals and empower the community to make informed health decisions. Given the potential of early diagnosis of MGUS and SMM to improve multiple myeloma treatment, we studied how to best share knowledge about multiple myeloma with the community and providers, as well as examine whether screening participants retained that knowledge.

## Patients and Methods

### Study design and setting

A multidisciplinary team designed and executed the study, including multiple myeloma specialists at the academic cancer center, a doctor of philosophy (PhD)–level cancer disparities researcher, a biostatistician, cancer center administrative leadership, and the director and program managers from the cancer center’s community outreach and patient navigation program. The community outreach team relied on relationships with community partners to execute health fairs and screening clinics in easily accessible public locations. The study developed multiple myeloma screening operations by modeling procedures on those already used by the community outreach program to navigate thousands of patients every year to breast, lung, prostate, and colorectal cancer screenings and address individuals’ barriers to care, including providing transportation, financial assistance, translation services, appointment coordination, and more. Study team members educated the public at community outreach screening events, and providers educated healthcare professionals at virtual continuing medical education (CME) events. The study was approved by the health system’s Institutional Review Board. Patient studies were conducted in accordance with the Belmont Report.

The study team used three modalities to administer the educational intervention. First, the creation and promotion of multiple myeloma screening events increased awareness among the public about the disease. Second, the outreach team distributed an original brochure to event attendees, whether they chose to participate in screening or not. Finally, the study team offered CME courses to community-based healthcare providers and healthcare professionals from several health systems to improve provider care for patients at high risk for multiple myeloma and encourage providers to involve their organizations in the screening events. A medical oncologist who specializes in multiple myeloma led two courses, and a PhD-level nurse practitioner in hematology and medical oncology led one course. Course topics included an overview of the disease, the benefits of early detection, and monitoring and treatment for patients diagnosed with MGUS, SMM, or multiple myeloma. The course reviewed who is at high risk for multiple myeloma, the multiple myeloma screening process, and the components of the M-protein test. Participants also discussed symptoms of multiple myeloma and its precursors that commonly lead to misdiagnosis.

### Study participants

An individual of any sex who identified as Black and/or African American and was at least 50 years of age was eligible to participate. To prioritize patient care for as many individuals as possible, the study used convenience sampling instead of a strategy for representativeness of the population. There was no randomization. Because this was a pilot study, a formal power calculation was not required. Participants did not preregister. To assist with study recruitment, organizations publicized events with flyers and announcements. After potential participants examined the educational brochure about multiple myeloma, they were given an opportunity to ask questions of the community outreach staff. Participants signed an informed consent document agreeing to a blood draw and to complete two surveys. Participants completed the study by completing the blood draw and the first survey; they could choose to skip questions or decline to complete the second survey; thus, there was no attrition.

The community outreach team identified providers and healthcare professionals for the CME course through convenience sampling, drawing on established relationships with federally qualified health centers, community organizations, and health systems. To assist with recruitment, individuals publicized the course with flyers and announcements that included details about the syllabus and the instructors who were multiple myeloma specialists from the academic cancer center.

### Intervention and data collection instruments

At the screening events, phlebotomists onsite drew participants’ blood, and community outreach personnel transported samples to the health system for processing. The blood test was the M-protein serum order in Epic, a single lavender top vacutainer tube with at least 3 mL collected to analyze free light chain levels, immunoglobulin levels, and serum immunofixation. Experienced providers reviewed all blood test results. Patient navigators notified participants of results via MyChart and phone calls. Patient navigators scheduled patients with abnormal results for follow-up and addressed barriers as needed.

In addition to the blood draw, participants completed an initial survey during the screening and a follow-up survey at least 1 week after the screening. Surveys addressed challenges with completing screenings, community reception to a multiple myeloma screening, and health literacy about multiple myeloma (see Table 1 for additional details about the data collection instruments). The study team developed the surveys by drawing on published surveys about cancer screenings (9, 17, 18, 22–26) and open-access national surveys, including the Health Information National Trends Survey (Med Ref Serv Q. 2021;40(2), RRID: SCR_023943), the Population Assessment of Tobacco and Health survey (US Department of Health and Human Services, Public-Use Files (ICPSR 36498) Version Date: Wave 5.5 (December 2019–November 2020), RRID: SCR_012945), and the Behavioral Risk Factor Surveillance System survey questionnaire (US Department of Health and Human Services, Centers for Disease Control and Prevention, 2019, RRID: SCR_012974). With permission, the study team utilized a question from the Barriers to Access to Care Evaluation scale to study barriers to care (27). As this was the first community-based multiple myeloma screening program, the study team also adapted questions from established breast cancer screening programs (28–31). The clinical team created questions about basic multiple myeloma knowledge. Participants completed four of the exact same knowledge questions on the initial and follow-up surveys. The study team adjusted scoring on a fifth question to account for a slight unforeseen formatting difference across the two surveys for this question only. This question asked participants to identify symptoms of multiple myeloma; a correct answer was recorded if a participant selected “all of the above” on the initial survey or if they selected each of these symptoms on the follow-up survey: tiredness, bone fractures without a known cause, and back pain. In addition, based on event observations, the study team collected information about screening location, reasons community members declined to participate, and strategies for effective recruitment. The team also conducted a chart review to record screening results and participants’ use of patient navigation.

**Table 1.** Data collection instruments.

Instrument	Who completed	Time completed	Content overview	Sample questions
Initial survey	Participant	During screening event	Demographic information Attitudes toward screening Multiple myeloma knowledge Patient experience and screening event feedback	Why did you decide to get a multiple myeloma screening? Choose the most appropriate response: Which of the following can be symptoms of multiple myeloma?
Event survey	Study team	Immediately following screening event	Event logistics Recruitment observations Information on those who declined to participate	Summarize techniques used to recruit patients and spark interest in the screening. Why did participants decline to participate?
Follow-up survey	Participant	At least 1 week after screening event	Multiple myeloma knowledge retention Patient experience and screening event feedback	How likely are you to recommend a friend or family member also participate in the multiple myeloma screening program?
Chart review datasheet	Study team	At least 1 week after screening event	Demographic information Screening result Patient navigation details	Barriers to care identified by the patient navigator (e.g., fear, transportation, financial aid, insurance, language/culture)

### Analysis

This article features the educational intervention and focuses on the multiple myeloma knowledge questions asked during the screening events and the follow-up survey. Community reception, more extensive analysis of blood draw results, event data, and patient navigation data are featured in other publications (32, 33). For the present analysis, the study team assessed multiple myeloma knowledge retention using descriptive statistics, including means, frequencies, and proportions, as well as the McNemar test to assess differences between the initial and follow-up surveys. To describe the healthcare professionals who attended CME courses, the study team collected data on attendees’ institutions, roles, and specialties. The study team assessed attendee data using descriptive statistics, including means, frequencies, and proportions. Study data were collected and managed using Research Electronic Data Capture (REDCap), a secure web-based software platform (REDCap Consortium, RRID: SCR_003445), and analysis was generated using Statistical Analysis System software (SAS Institute Inc., 2023, version 9.4; RRID: SCR_008567).

## Results

### Multiple myeloma screening

A total of 199 individuals participated in 22 multiple myeloma screening events from June 2023 to December 2024 at community organizations and churches in a large Midwest city. Participants identified as Black and/or African American and multiracial. The median age was 66 years. Most participants identified as female (*n* = 166, 83%), and some reported a family history of multiple myeloma (*n* = 24, 12%; see Table 2 for additional demographic characteristics). Twenty-nine individuals (15%) received abnormal blood draw results; upon provider review, 21 individuals (11%) were diagnosed with MGUS, with no cases of multiple myeloma or SMM. Patients diagnosed with MGUS reflected the sample, with 81% identifying as female and a median age of 66 years.

**Table 2.** Participant demographic characteristics (*N* = 199).

Characteristic	*n*	Percentage (%)
Age	​	​
50–60	64	32
61–74	112	56
75+	23	12
Sex	​	​
Woman	166	83.4
Man	33	16.6
Sexuality	​	​
Straight/heterosexual	112	56
Other sexuality or no response^a^	87	44
Race	​	​
Black and/or African American and multiracial^a^	199	100
Ethnicity	​	​
Non-Hispanic	196	98
Hispanic or no response^a^	3	2
Marital status	​	​
Married	71	36
Other or no response	128	64
Education	​	​
High school or associate’s degree	17	8.5
College/bachelor’s degree	26	13.1
Advanced degree (master’s/doctorate)	14	7
No response	142	71.4
Income	​	​
<$10,000	9	4
$10,000–$24,999	18	9
$25,000–$49,999	14	7
$50,000–$99,999	19	10
$100,000 or more	20	10
Prefer not to answer or no response	119	60
Insurance	​	​
Medicaid	7	4
Medicare	40	20
Private	52	26
Other	3	2
No response	97	48
Have a regular doctor and/or a PCP	​	​
Yes	141	71
No, I do not know, or no response	58	29
Family history of multiple myeloma	​	​
Any family member	24	12
No one has multiple myeloma	94	47
I do not know or no response	81	41
Previously completed another cancer screening with the outreach team	42	21
One barrier to care (scheduling, financial, or health literacy)	101	51
Two barriers to care (scheduling, financial, and/or health literacy)	5	2
No barriers to care or no response	93	47
Abnormal results	​	​
No	170	85
Yes	29	15
MGUS diagnosis	21	11

a Responses aggregated to protect confidentiality.

All abnormal results were reviewed by a hematologist prior to an advanced practice provider reporting these results to patients. All of those diagnosed with MGUS received follow-up care from the multiple myeloma group. Testing included serum/urine protein electrophoresis, complete blood count, comprehensive metabolic panel, whole-body bone imaging with either skeletal survey or whole-body low-dose computed tomography scan, and potentially a bone marrow biopsy. The cadence for future follow-up included annual monitoring with lab testing and bone imaging. Among the 21 patients found to have MGUS, most were IgG MGUS. In addition, eight patients’ blood draw results were abnormal but did not indicate MGUS. These patients had either autoimmune disease or a very slightly elevated light chain level that was marked as abnormal but was not considered MGUS. An advanced practice provider discussed their blood work and other conditions that would lead to these results and then informed patients that their results were actually normal.

The reactions of participants who may have experienced unnecessary worry are of particular interest in a study designed to evaluate both the positive and negative implications of screening for multiple myeloma. Among participants with abnormal blood results who were not ultimately diagnosed with MGUS, seven of eight (87.5%) completed a follow-up survey. All seven reported that on a scale of 1 to 100, participating in the screening program was rated 1 at the easiest level (1 being very easy and 100 being very difficult to participate). Likewise, on a scale of 1 to 100, all seven rated their likelihood to recommend a similar program to a friend or family member at 100, the highest level (1 being very unlikely and 100 being very likely to recommend to a friend or family member). Reported in a separate publication, the majority of participants in the screening program overall also rated participation as very easy, with a high likelihood of recommending a similar program (32, 33).

### Provider training

Forty-six healthcare professionals participated in three courses. Half of the participants were employed at the academic medical center sponsoring this study (*n* = 23, 50%), and half were employed at other facilities (*n* = 23, 50%). Participants identified as physicians, physician assistants, residents or fellows (*n* = 15, 32.6%), nurses or advanced practice nurses (*n* = 8, 17.4%), and a variety of other health professionals. Additional details about participants are described in Table 3.

**Table 3.** CME event attendees (*N* = 46).

Characteristic	*n*	Percentage (%)
Institution	​	​
Employed at sponsoring academic hospital	23	50
Employed elsewhere	23	50
Role	​	​
Physician	11	23.9
Physician assistant	2	4.3
Resident/fellow	2	4.3
Nurse	5	10.9
Advanced practice registered nurse	3	6.5
Corporate	4	8.7
Pharmacist	2	4.3
Allied health professional/social worker	1	2.2
Researcher	2	4.3
Other	14	30.4
Specialty	​	​
Internal medicine/family practice	12	26
Hematology/oncology	11	23.9
Nursing	4	8.7
Education	3	6.5
Other/none	16	34.8

Fourteen participants chose to claim CME credits and therefore completed an assessment of the course. The majority of participants gave the course the highest rating, with 85.7% (*n* = 12) expressing that the program was excellent. The program was also seen as impactful, with 71.4% of participants (*n* = 10) noting that the course would very likely change their practice behavior. In an open-ended question, participants explained they intend to change their practice by better recognizing high-risk patients, conducting multiple myeloma screening, and educating their patients and colleagues about multiple myeloma. See Table 4 for additional details.

**Table 4.** CME evaluation assessment.

Evaluation question	*n*	Percentage (%)
What was your overall opinion of the activity? (Excellent, Good, Average, Fair, Poor)	​	​
Excellent	12	85.7
Good	2	14.3
Please rate the degree the activity impacted you. (High, Moderate, No)	​	​
High impact	10	71.4
Moderate impact	4	28.6
The activity increased my knowledge. (Strongly Agree, Agree, Disagree, Strongly Disagree)	​	​
Strongly agree	13	92.9
Agree	1	7.1
The activity increased my competence. (Strongly Agree, Agree, Disagree, Strongly Disagree)	​	​
Strongly agree	8	57.1
Agree	6	42.9
The activity will improve my performance. (Strongly Agree, Agree, Disagree, Strongly Disagree)	​	​
Strongly agree	7	50
Agree	7	50
This activity will help me be a more effective member of the healthcare team. (Strongly Agree, Agree, Disagree, Strongly Disagree)	​	​
Strongly agree	10	71.4
Agree	4	28.6
This activity will help me improve patient care. (Strongly Agree, Agree, Disagree, Strongly Disagree)	​	​
Strongly agree	9	64.3
Agree	5	35.7
Based on this activity, will you change your practice behaviors? (Very Likely, Likely, Somewhat Likely, Not at All, Not Applicable)	​	​
Very likely	10	71.4
Likely	2	14.3
Somewhat likely	2	14.3
How will you change your practice? (Select all that apply)		
Apply scientific information to change the management/treatment of patients.	9	64.3
Communicate better with patients, their families, and the healthcare team.	6	42.9
Adhere to professional practices and ethical principles.	3	21.4
Coordinate care/utilize resources within the healthcare system.	4	28.6
Interact with patients more collaboratively to promote health and address diverse needs.	4	28.6
Improve the quality and safety of care.	3	21.4
Utilize information technology more effectively.	2	14.3
Work more effectively as a member of the healthcare team.	5	35.7
I will not make any changes to my practice.	1	7.1

### Brochure

**Figure 1.**
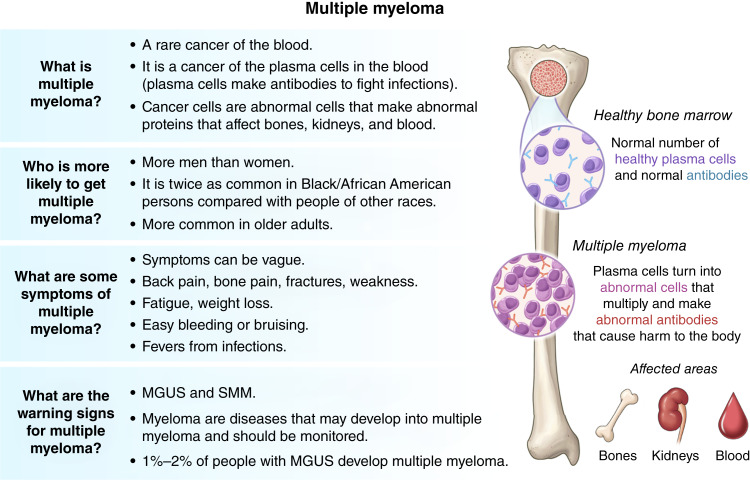
Educational brochure. The study team provided this brochure to community members who attended screening events. The brochure describes multiple myeloma, those at risk, and the symptoms and warning signs, as well as an infographic of the disease.

Upon recruitment to the study, participants were provided with an educational brochure designed to address multiple myeloma in Black populations (see Fig. 1). The brochure explained multiple myeloma, the disease process, multiple myeloma’s prominence in the African American community, and symptoms of the disease. After reviewing the brochure and completing the blood draw, participants answered five questions that corresponded to the information in the brochure.

### Knowledge retention

After reviewing the brochure, participants answered a series of questions about multiple myeloma and its precursors. Facts about the African American community resonated best with participants, with 79.2% of participants (*n* = 152) correctly noting that multiple myeloma is twice as common among African Americans as compared with other racial groups. Slightly less than two thirds of the participants were also able to define multiple myeloma as a rare cancer of the blood (*n* = 121, 63%) and identified symptoms of multiple myeloma (*n* = 123, 64.7%). Fewer correctly identified the function of cancerous plasma cells (*n* = 104, 54.7%) or that individuals diagnosed with MGUS have an abnormal protein in the blood commonly referred to as M-protein (*n* = 59, 30.9%).

After participants received their multiple myeloma screening results, a study team member called at least 1 week following the initial screening to reexamine multiple myeloma knowledge and reception to the screening. The study team reached 164 of 199 (82.4%) participants to complete the follow-up survey. Of 199 participants, 158 completed the knowledge surveys both at the screening event and during the follow-up call (79.3%). Among the 158 individuals who completed both the initial and follow-up surveys, the percentage of correct responses increased on all items. Correct responses increased significantly on items about multiple myeloma being a rare cancer of the blood (19%, *P* < 0.0001) and individuals with MGUS having an abnormal protein in the blood (12.1%, *P* = 0.002). See Table 5 for additional details. The increases in the scores suggest that participants retained the knowledge about multiple myeloma that they gained during their participation in screening.

**Table 5.** Knowledge of multiple myeloma at the time of intervention and follow-up call.

Knowledge question	Immediately after intervention completed *N* [Correct *n*] (Percent correct)	At follow-up completed *N* [Correct *n*] (Percent correct)	Change among 158 participants who completed both surveys Correct increase % *P* value
Is the following statement true or false? Multiple myeloma is a rare cancer of the blood.	192 [121] (63%)	164 [137] (83.5%)	19% *P* < 0.0001
Is the following statement true or false? Multiple myeloma is twice as common among African Americans as other racial groups.	192 [152] (79.2%)	164 [142] (86.6%)	7% *P* = 0.07
Which of the following can be symptoms of multiple myeloma? (Tiredness, Bone fractures without a known cause, Back pain, or All of the above)	190 [123] (64.7%)	164 [113] (68.9%)	3.2% *P* = 0.46
Monoclonal gammopathy of undetermined significance, known as MGUS for short, is a disease that patients may get before multiple myeloma. MGUS can be found with a simple blood test. Which of the following is true about individuals who have MGUS? They have an abnormal protein in their blood called M protein. They never develop multiple myeloma. They almost always experience precancerous symptoms. I don’t know.	191 [59] (30.9%)	164 [75] (45.7%)	12.1% *P* = 0.002
Multiple myeloma is a cancer of the plasma cell in the blood. What do these cancer cells do to the body? The disease can make you feel extremely lively and energetic. The cancer cells can damage your skin and ears. The cancer cells often cause damage to your bones and/or kidneys. I don’t know.	190 [104] (54.7%)	164 [93] (56.7%)	5.8% *P* = 0.44

The intervention left participants with similar or higher levels of multiple myeloma knowledge regardless of their demographic characteristics and previous experience with multiple myeloma. Slight variations from the mean were found, with men, those with a family history of multiple myeloma, individuals working part-time, and those with a high school level of education demonstrating slightly higher than average scores on multiple myeloma knowledge. It is important to note, though, that the very limited number of participants in some of these categories suggests that these differences are likely due to chance. For example, there are only six participants who reported a high school level of education and six participants who reported working part-time. Regardless of these small differences overall, the educational materials were effective across demographic characteristics.

## Discussion

We developed and executed a novel multiple myeloma screening program that educated participants, community members, and local healthcare professionals. The clinical team diagnosed 21 (11%) of screened individuals with MGUS, and all received follow-up testing and plans for surveillance. Current research supports better survival when multiple myeloma is treated prior to end-organ damage, bone destruction, and renal failure (9).

Compared with existing research about MGUS incidence in the general population (8, 34), our study results are higher than those previously reported, except among Black US veterans (34, 35). However, 83% of participants in this study identified as female, and literature suggests that MGUS is more prevalent among males (19). Our disproportionately female sample may have resulted in fewer MGUS diagnoses than could be found by screening more men. Men are less likely to participate in preventative medicine generally and cancer screening specifically (36). With greater male participation in multiple myeloma screening programs, research suggests MGUS rates in a Black population could be as high as 25% (9). Results suggest an urgent public health need for multiple myeloma screening for high-risk populations. Future research should continue to implement and evaluate screening programs for individuals at high risk for multiple myeloma and its precursors, such as Black individuals above the age of 50, especially men, and individuals with a family history of multiple myeloma.

This study provides a model for how to share normal and abnormal multiple myeloma screening results with community members. Participants overall rated the program highly, even among those who received abnormal results but were not diagnosed with MGUS. These results suggest that education about multiple myeloma can be more empowering than distressing, with the support of a multidisciplinary team that includes hematologists, patient navigators, and an advanced practice provider experienced in providing healthcare to underserved populations. Further studies should assess this possibility in a larger sample.

Participants were somewhat knowledgeable at the time of screening and retained that knowledge past the moment of participation. Slightly higher levels of knowledge after time passed suggest that participants may have even done their own research to supplement the knowledge shared by the study. It is also possible that the 22 community-wide screening events and three CME courses have started a community-wide conversation about multiple myeloma that will help maintain knowledge of and interest in this disease.

Key findings from this pilot that may inform future projects include employing a multidisciplinary team to offer education to several groups, which contributed to driving community conversations and screening participation. About half of the participants listed a clinical field (physicians, nurses, pharmacists, social workers) that may have a direct opportunity to affect health decisions. The nonclinical participants can also educate the community about multiple myeloma; for example, informed community leaders can encourage screening or recognize symptoms in their acquaintances and advocate for testing.

Perhaps the most important lesson learned is that effective research and health interventions are accomplished with community partnerships. Outreach targeted community leaders from local organizations and churches, in addition to community-based PCPs. Recruiting participants relied on long-term trusting relationships to influence the spread of information and the willingness of organizations to host screening events, instead of recruiting established patients identified by electronic medical records or those visiting a medical center. If healthcare professionals and advocates truly want to screen a high-risk Black population, programs must have the support of community leaders, go onsite in the community, educate the target population about screening, and then secure informed consent and obtain blood tests in the community as well. Future studies aiming to identify high-risk populations should consider this type of broad recruitment strategy and wide-reaching educational intervention.

The comprehensive nature of this intervention targeted a wider scope of influence than can be measured purely through individual-level knowledge scores. Further research would be helpful to better understand the longer-term changes in health literacy about multiple myeloma overall as the community, providers, and patients are exposed to robust interventions that may alter thinking around preventative healthcare. These results help support the efficacy of a screening program for high-risk populations, especially Black individuals (4). Black individuals were receptive to a screening program that educated their community about multiple myeloma disparities. More so than other components of the educational materials, participants remembered that there were higher multiple myeloma risks for Black individuals, and they retained that knowledge when contacted at a later date. It is likely that communicating the specific risks for the Black community inspired high levels of participation, and similar educational campaigns could encourage high levels of participation in future studies.

### Limitations

We conducted screenings at one point in time, in one Midwest city, and with a small cohort of Black individuals aged 50 and older. This is a limited population gathered with convenience sampling to maximize screenings to community partner organizations, without sampling for representativeness, and our sample is disproportionately female, straight/heterosexual, Black, and non-Hispanic. Especially given higher rates of MGUS among men than women (36), it is important that future studies outreach specifically to men, such as by educating them about their higher risk.

In terms of assessing knowledge about multiple myeloma, the study team provided the original brochure during recruitment at the screening events; thus, we were unable to determine knowledge levels prior to study exposure. Without an assessment prior to study exposure, the effectiveness of educational materials or the surveys’ validity could not be fully determined and is an objective for future research. Also, several individuals declined to answer the knowledge questions on the first survey (*n* = 7, 4%), could not be reached or declined to answer the follow-up survey (*n* = 35, 18%), or skipped some of the questions, further limiting our sample size.

Future research should consider addressing specifically the symptoms of multiple myeloma and providing education that helps participants identify these symptoms. Also, further research is needed to examine whether community members and study participants who learned about multiple myeloma as part of this study are more likely to address multiple myeloma with their PCPs. With respect to the education provided to healthcare professionals in the CME course, future studies should examine comprehension among all participants, even those who do not claim CME credit, and investigate whether the training affected healthcare professionals’ work in clinical and community settings. Additional follow-up is warranted to examine whether providers who attended the CME course actually changed their clinical practice toward more multiple myeloma screening and education among their patients after the course.

A comprehensive intervention that combines education and screening yields retention of knowledge about multiple myeloma. Combining educational opportunities with community-based screening events encourages participation among Black individuals over 50 years old. This study reveals that community members are receptive to multiple myeloma screening when healthcare professionals communicate with them about their risks and share knowledge about disease symptoms and incidence. Screening programs that meet high-risk populations in their community, intervene at multiple levels, and educate people about their risk can be effective ways to identify individuals likely to benefit from multiple myeloma screening.

## Data Availability

The data generated in this study are available from the corresponding author upon request.
